# Impact of Social Determinants of Health, Health Literacy, Self-perceived Risk, and Trust in the Emergency Physician on Compliance with Follow-up

**DOI:** 10.5811/westjem.2020.12.48981

**Published:** 2021-05-05

**Authors:** James Sutton, Leon Gu, Deborah B. Diercks

**Affiliations:** * University of Texas Southwestern School of Medicine, Dallas, Texas; † University of Texas Southwestern, Department of Emergency Medicine, Dallas, Texas

## Abstract

**Introduction:**

Patients presenting to the emergency department (ED) with “low-risk” acute coronary syndrome (ACS) symptoms can be discharged with outpatient follow-up. However, follow-up compliance is low for unknown nonclinical reasons. We hypothesized that a patient’s social factors, health literacy, self-perceived risk, and trust in the emergency physician may impact follow-up compliance.

**Methods:**

This was a prospective study of a convenience sample of discharged ED patients presenting with chest pain and given a follow-up appointment prior to departing the ED. Patients were asked about social and demographic factors and to estimate their own risk for heart disease; they also completed the Short Assessment of Health Literacy-English (SAHL-E) and the Trust in Physician Scale (TiPS).

**Results:**

We enrolled146 patients with a follow-up rate of 36.3%. Patients who had a low self-perceived heart disease risk (10% or less) were significantly less likely to attend follow-up than those with a higher perceived risk (23% vs 44%, P = 0.01). Other factors did not significantly predict follow-up rates.

**Conclusion:**

In an urban county ED, in patients who were deemed low risk for ACS and discharged, only self-perception of risk was associated with compliance with a follow-up appointment.

## INTRODUCTION

Chest pain is a most commonly presenting symptom in the emergency department (ED).^1^ One of the greatest concerns for chest pain patients is acute coronary syndrome (ACS), which includes high-mortality issues such as myocardial infarction. ED discharge is appropriate if a patient’s history, electrocardiogram, troponin levels, and other risk factors are considered low risk.^2^,^3^ However, follow-up is recommended even if appropriately discharged.^4^ Follow-up compliance in these discharged patients is low, with only 70% attending primary care follow-up within 30 days.^2^–^5^ The American Heart Association/American College of Cardiology recommend follow-up within 72 hours of discharge, a guideline that has compliance as low as 6%.^4^ Previous research has shown that social and demographic factors such as health insurance and socioeconomic status may impact outcomes and follow-up.^2^,^3^

In addition, previous research has indicated that an appointment scheduling system and health insurance are high-yield targets to improve patient follow-up.^3^,^6^ Little is known about subjective variables such as self-perceived risk for heart disease, trust in the emergency physician, comfort with diagnosis, and health literacy. The objective of this study was to determine whether these factors have an impact on follow-up.

## METHODS

This was a prospective study of a convenience sample of patients discharged with follow-up after a visit for chest pain. This study was reviewed and approved by our institutional review board. The population included those given acute response clinic (ARC) appointments following ED discharge from an urban hospital in Dallas between November 2017–March 2019. Eligible patients were English-speaking, older than 18 years, and presented with chest pain, later determined to be low risk for ACS. Patients had to be referred to an ARC appointment before being discharged. Exclusion criteria included pregnant patients, prisoners, homeless patients, or those with human immunodeficiency virus (HIV). Homeless patients and those with HIV are referred to separate clinics that specialize in holistic care for these populations. We excluded these populations in order to isolate patients referred exclusively for chest pain.

Acute response clinic appointments are available for local county residents as a way to receive primary care follow-up. If the resident has an established provider, an appointment is scheduled with that provider instead. Appointments to the ARC are made by case management staff and reviewed with the patient before discharge. An author verified that an ARC appointment within 30 days was provided before enrolling patients.

Eligible patients were enrolled in person before discharge using a pre-assembled study packet. Information was acquired verbally after obtaining consent. Studied demographic information included gender, age, race, ethnicity, and religious affiliation. Social determinants of health (SDH) is defined as the conditions in which people are born, grow, live, work and age, as well as the drivers of those conditions.^3^ Socioeconomic factors in particular, such as income, education, and employment, are major influences.^3^ Therefore, we chose to evaluate education, employment, marital status, household income, and insurance status as primary SDH. An income of $10,000 a year was set as the cutoff for household income, as a simplified means of identifying poverty.

Patients were asked, “What do you think your risk for heart disease is with 0% being no risk and 100% being certain you have heart disease?” This question was repeated as often as necessary without further clarification. Low-risk patients had a self-perceived risk of 0–10%, high-risk patients 11–99%, and certain patients 100%. We chose 10% as the low-risk cutoff based on tools such as the Framingham risk score for hard coronary heart disease and the prospective cardiovascular Munster study (PROCAM) risk calculator, which estimate 10-year cardiovascular disease risk. Framingham scores based on Adult Treatment Panel III classify men as low risk if their 10-year risk of cardiovascular events is <10%, and PROCAM also classifies scores <10% as low risk. Thus, we determined 10% was an appropriate “low-risk” cutoff.^7^

Patients were given the Short Assessment of Health Literacy-English (SAHL-E) to determine health literacy; a score of 14 or lower on the 18-item exam determined low health literacy.^8^ Health literacy is associated with adherence, especially for non-medication regimens and cardiovascular disease.^9^ A visual analogue scale for discomfort, based on a Likert scale, was used to determine comfort level with their ED diagnosis, composed of large numbers from 0–5 with “0” representing total satisfaction and “5” representing complete discomfort.^10^ Below these numbers was the request “rate comfort level with diagnosis.” Finally, the Trust in Physician Scale (TiPS) was given to determine the level of trust in the patient’s emergency physician.^11^ Trust in physician is correlated with continuation of care.^12^ We separated the TiPS scores into tertiles, representing low, medium, and high trust.

We assessed barriers to follow-up, including transportation, cost of parking, getting time off work, not understanding why the appointment was made, family obligations, and appointment length, Primary outcome was attendance at follow-up appointment. Using the patient’s electronic health (EHR), we reviewed whether or not they attended their ARC follow-up or used another provider.

### Analysis Plan

All variables were categorical and are presented as the count and percent frequency of occurrence. Patients who attended their follow-up appointments were compared to those who did not with regard to the above variables using either a chi-square test or a Fisher’s exact test, as appropriate. Then, to account for confounders we performed a multivariate logistic regression analysis to determine whether any of the above variables were associated with successful follow-up. All *P*-values are two-sided and considered significant at the 5% level. Analysis was done using SOFA Statistics software (Paton-Simpson & Associates Ltd, Auckland New Zealand) and R software (the R Project for Statistical Computing, Vienna, Austria) [Figure].

## RESULTS

Overall, approximately 10% of eligible, English-speaking patients who were given an ACR appointment after being discharged from the ED due to chest pain were captured. We enrolled 146 patients: 47 (32.2%) showed for their ARC appointments and 82 (56.2%) failed to show despite having an appointment. Seventeen (11.6%) patients cancelled their initial ARC appointment, with two (1.7%) attending another ARC appointment; four patients (2.7%) achieved outside follow-up. Overall, 53 (36.3%) patients achieved some form of follow-up. No demographic factor was associated with ARC follow-up rate.

Of the 53 patients reporting low risk of heart disease (self-assessed risk <10%), only 12 showed for their appointment (22.6%). The 67 patients reporting high risk (self-assessed risk 11–99%) showed for their appointment 44.8% of the time, and those who were certain they had heart disease showed 42.3% of the time. Patients who considered themselves to be at low risk were less likely to attend their follow-up appointments than those who considered themselves to be at high risk or certain (22.6% vs. 44.1%, *P* = 0.01) [Table 1].

We identified 29.5% of patients as having low health literacy. The majority (52.7%) of patients were comfortable with their ED diagnoses, and most (77.1%) trusted their emergency physician. Table 2 shows assessed SDH as well as patient health literacy, their comfort with their diagnosis, and their TiPS score. No SDH was significantly associated with follow-up. We found no significant association between the other variables and show rate.

The majority of our patients reported at least one barrier to follow-up (54.8%). Although 42.5% of patients who reported one or more barriers showed for their ARC appointment compared to 28.8% of patients reporting no barrier attending, there was not a significant association (*P* = 0.09). Of those, the majority (52.5%) of patients reported transportation as a potential barrier. Table 3 details which barriers in particular were reported.

Following initial univariate analysis with chi-square, we performed a multivariate logistic regression analysis, the results of which are detailed in Table 4. Self-perceived risk remains the only variable significantly associated with follow-up rate.

## DISCUSSION

Outpatient follow-up is critical to manage patients at low risk for ACS after ED discharge. Compliance in our population is low at 36.3%, relative to previous studies with compliance around 65%.^2^ As this number does not include the large population with a pre-existing primary care provider, we could not determine whether this was secondary to included factors or to a different population. However, we did expect higher levels given our appointment-setting protocol.^2^

Importantly, our study showed that self-perceived heart disease risk is associated with follow-up. Social determinants and health literacy were not associated with follow-up, implying education is not the primary factor. Although the SAHL-E and TiPS have good reliability and validity,^8^,^11^ to our knowledge TiPS has not yet been validated in an emergency setting, and is intended for outpatient clinic assessment. There is a fundamental shift in provider between ED and clinic, and the impact of having trust in a physician who is not managing continuing care has yet to be seen.

Previous research has shown that patients cannot accurately report their own cardiovascular risk despite accurately reporting risk factors, with almost 90% of patients underestimating their risk. Patients are often unable to relate their risk factors with actual risk for cardiovascular events.^13^ Prior events such as previous emergency assessments for chest pain, strong family history for ACS, and medical history including risk factors such as hypertension and diabetes may influence patients to have a higher self-perceived risk. Stressing actual risk of cardiovascular events with patients, potentially using objective assessment tools such as PROCAM and Framingham, may be helpful for emergency physicians to adjust self-perceived risk to be more in line with actual risk and in turn improve follow-up rates. It may be difficult to apply this to patient care to improve follow-up. Health education is likely not sufficient as an intervention; health literacy and education status were not significantly associated with show-up rates. However, cardiovascular-specific education and individualized education has previously proven to be helpful.^13^

## LIMITATIONS

This study has a few limitations to consider. As we required an ARC appointment to be eligible, patients referred to other clinics or an existing provider were excluded. We also excluded Spanish-speaking only patients who are a significant portion of the hospital population. Of the patients who potentially qualified, only a relatively small percentage (10.8%) of them could be interviewed primarily due to limited data collector availability. There was only one active interviewer at a time, and interviewers were not necessarily available on a regular basis. This further limitation resulted in wider than desirable confidence intervals. We did not inquire about primary care follow-up beyond use of the EHR. Although the hospital-associated system includes many providers, it is possible that patients attended follow-up out of network. Overall, this study is generalizable to urban institutions that care mostly for low-income patient populations. It is unclear how institutions that provide for different patient populations would be impacted.

Additional limitations involve questionnaire validity. It is possible that our question may have been interventional; by asking patients to self-assess, they may have become more inclined to follow up. Our question was not validated by other studies. The lack of detail included in the question may have been confusing, as patients may not have understood whether risk meant heart failure, coronary artery disease, or other heart-related diseases and issues. In addition, the visual analogue scale used to determine comfort with diagnosis was not a previously studied or validated scale.

## CONCLUSION

We found that self-perceived risk for heart disease is associated with follow-up rates in patients who present to the ED with chest pain. We failed to find an association between social determinants of health, health literacy, trust in physician or barriers to access, and follow-up rates in these patients. Conversations with patients about their actual risk of ACS, such as with objective cardiovascular risk assessments such as the PROCAM and Framingham tools, may improve patient compliance with follow-up. Future studies should investigate how to improve follow-up compliance.

## Figures and Tables

**Figure:**
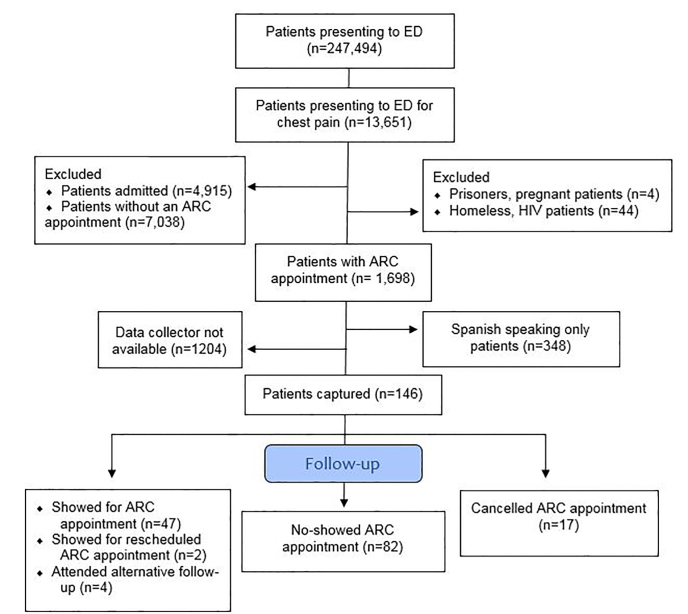
CONSORT diagram. Flow diagram showing process for patient selection and exclusion for the study. *ED*, emergency department; *ARC*, acute response clinic; *HIV*, human immunodeficiency virus.

**Table 1:** Comparison between patient self-perceived risk for heart disease, total number of patients in each self-perceived risk category, and the number of patients who showed up for their acute response clinic appointment.

Self-perceived risk	Participants, n(%)	Show number and rate (%)
Low	53 (36.3)	12 (22.6)
High	67 (45.9)	30 (44.8)
Certain	26 (17.8)	11 (42.3)

**Table 2:** Social determinants of health (SDH), health literacy, emergency department (ED) diagnosis comfort, and Trust in Physician TiPS scale. Comparison between patient variables, number of participants in each variable, and show rate for those participants. Show rate difference is the largest difference in percentages between overarching variables. No SDH was significantly associated with follow-up. No significant association between other variables and show-rate was found.

Variable	Participants n, (%)	Show number and rate (%)	Show rate (%) difference	95% CI (%)
Education	146		11.1	−6,28
High school graduate or higher	93 (63.7)	30 (32.3)		
Non-graduate	53 (36.3)	23 (43.4)		
Employment	145		4.2	−13,20
Yes	56 (38.4)	19 (34.0)		
No	89 (61.6)	34 (38.2)		
Marital status	143		3	−16,23
Married	33 (23.1)	13 (39.4)		
Not married	110 (76.9)	40 (36.4)		
Health insurance status	146		8.7	−9,24
Uninsured	55 (37.7)	17 (30.9)		
Insurance or discount program	91 (62.3)	36 (39.6)		
Household income	127		12.6	−11,26
< $10,000/year	40 (31.5)	17 (42.5)		
> $10,000/year	87 (68.5)	26 (29.9)		
Health literacy	146		4.5	−13,23
Low health literacy	43 (29.5)	17 (39.5)		
Normal health literacy	103 (70.5)	36 (35.0)		
Diagnosis comfort	146		3.6	−17,22
Comfortable	77 (52.7)	27 (35.1)		
Mildly uncomfortable	38 (26.0)	14 (36.8)		
Very uncomfortable	31 (21.2)	12 (38.7)		
TiPS scale	146		12.8	−12,34
Low	48 (32.9)	21 (43.8)		
Medium	69 (47.3)	23 (33.3)		
High	29 (19.8)	9 (31.0)		

*CI*, confidence interval; *TiPS*, Trust in Physician Scale.

**Table 3:** Reported barriers to follow- up. Compares barriers reported and the number of participants reporting each barrier.

No barriers	Transportation	Time of appt	Cost of parking	Time off work	Don’t understand appt need	Family obligations	Length of appt
66	42	25	25	15	3	15	7

*Appt*, appointment.

**Table 4:** Low vs high/certain self-perceived risk was significantly (P = 0.01) associated with follow-up rates when using multivariable logistic regression to account for confounding. No other variable was found to be significantly associated.

Variable	P-value	Odds ratio	95% confidence interval
Self-perceived risk	0.01	2.84	(1.25,6.42)
Gender	0.62	0.82	(0.39,1.74)
Race	0.68	0.95	(0.75,1.20)
Ethnicity	0.19	1.66	(0.77,3.54)
Religion	0.47	1.00	(0.99,1.00)
Education level	0.67	1.00	(0.98,1.02)
Employment	0.86	0.93	(0.42,2.03)
Marital status	0.64	0.99	(0.96,1.02)
Income	0.66	0.99	(0.99,1.00)
Insurance	0.26	1.07	(0.94,1.22)
Health literacy	0.20	1.72	(0.74,4.01)
TiPS	0.32	0.76	(0.44,1.30)
Barriers	0.21	1.63	(0.74,3.57)
VAS	0.91	0.97	(0.61,1.55)
Constant	0.01		

*TIPS*, Trust in Physician Scale; *VAS*, visual analogue scale.
